# Using a Material Library to Understand the Change of Tabletability by High Shear Wet Granulation

**DOI:** 10.3390/pharmaceutics14122631

**Published:** 2022-11-28

**Authors:** Yawen Wang, Junjie Cao, Xiaoqing Zhao, Zichen Liang, Yanjiang Qiao, Gan Luo, Bing Xu

**Affiliations:** 1 Department of Chinese Medicine Informatics, Beijing University of Chinese Medicine, Beijing 100029, China; 2 Beijing Key Laboratory of Chinese Medicine Manufacturing Process Control and Quality Evaluation, Beijing 100029, China

**Keywords:** change of tabletability, high shear wet granulation, material library, tabletability change classification system, critical material attributes

## Abstract

Understanding the tabletability change of materials after granulation is critical for the formulation and process design in tablet development. In this paper, a material library consisting of 30 pharmaceutical materials was used to summarize the pattern of change of tabletability during high shear wet granulation and tableting (HSWGT). Each powdered material and the corresponding granules were characterized by 19 physical properties and nine compression behavior classification system (CBCS) parameters. Principal component analysis (PCA) was used to compare the physical properties and compression behaviors of ungranulated powders and granules. A new index, namely the relative change of tabletability (*CoT*_r_), was proposed to quantify the tabletability change, and its advantages over the reworking potential were demonstrated. On the basis of *CoT*_r_ values, the tabletability change classification system (TCCS) was established. It was found that approximately 40% of materials in the material library presented a loss of tabletability (i.e., Type I), 50% of materials had nearly unchanged tabletability (i.e., Type II), and 10% of materials suffered from increased tabletability (i.e., Type III). With the help of tensile strength (TS) vs. compression pressure curves implemented on both powders and granules, a data fusion method and the PLS2 algorithm were further applied to identify the differences in material properties requirements for direct compression (DC) and HSWGT. Results indicated that increasing the plasticity or porosity of the starting materials was beneficial to acquiring high TS of tablets made by HSWGT. In conclusion, the presented TCCS provided a means for the initial risk assessment of materials in tablet formulation design and the data modeling method helped to predict the impact of formulation ingredients on the strength of compacts.

## 1. Introduction

Nowadays, tablets represent the most widely utilized oral solid dosage (OSD) form in drug manufacturing due to the advantages of self-administration, stability, ease of handling, transportation, and good patient compliance [1]. According to the US Food and Drug Administration (FDA) Center for Drug Evaluation and Research (CDER) report on novel drug approvals, tablets accounted for 39.6%, 26.4%, and 30.0% of new pharmaceutical products for three consecutive years from 2019 to 2021, respectively, showing that tablets were the first choice of OSD forms in the drug development process [2,3,4]. The basic manufacturing routes of tablets mainly include direct compression (DC), wet granulation (WG), dry granulation (DG), and other technologies. DC is a well-known and simple method in tablet manufacture due to the saving of time, labor, and cost, but it has high requirements and limitations on physical properties of raw materials, such as flowability, compactability, and die filling of materials [5]. In practice, it is often necessary to convert fine powders into large agglomerates by granulation processes to improve the compaction and flow properties of the material. According to the research on the manufacturing classification system (MCS), 16% of commercial tablet formulations were manufactured using the direct compression process. Meanwhile, 52% of the commercial tablet formulations were produced by the granulation process, in which 77% were from wet granulation [6]. This showed that WG was the most popular process option followed by DC and DG, since WG had a higher tolerance for poor physical properties of materials.

The tensile strength (TS) of a tablet is an important quality attribute as the tablet needs to be mechanically strong to withstand further handling, such as film-coating, packaging, transport, and end use by the patient [7]. However, a common problem that cannot be ignored in granulation is the loss of tabletability or loss of reworkability, which has a direct effect on tablet tensile strength [8]. At present, the effects of dry granulation on the tabletability of pharmaceutical materials have been thoroughly investigated. Since the material underwent two times of compression, i.e., rolling and tableting, the granules often showed significant loss of tabletability compared with raw powders [9]. The research on reduced tabletability during dry granulation mainly focused on two aspects. The first aspect was to study the mechanisms of loss of tabletability. There were mainly two well-recognized hypotheses about the mechanisms of reduced tabletability in dry granulation, i.e., the granule hardening and the granule size enlargement [10]. For plastic materials, the loss of tabletability in dry granulation was mainly related with both the granule size enlargement and the granule hardening [11,12,13]. For brittle materials, the granule hardening had a greater impact on loss of tabletability than other mechanisms [14,15,16]. The second aspect was to investigate the factors that affected the reduced tabletability of materials after DG, such as physical properties of raw materials (i.e., particle sizes, particle morphology, particle porosity and moisture content) [17,18,19,20], ribbon properties (i.e., porosity and tensile strength) [21,22,23], process parameters of roll compaction and dry granulation (i.e., rolling pressure, speed of screw and roll, milling time and sieve size) [9,24,25,26], and tableting process parameters (i.e., lubricant usage and dosage, tableting pressure and tableting speed) [27,28,29]. Compared with brittle materials, formulations dominated by plastic materials were more sensitive to process parameters and were more prone to loss of tabletability [10]. It was worth noting that some brittle materials, e.g., acetames drug levetiracetam, had increased tabletability after dry granulation, which was related to the increase in specific surface area of this drug [30]. The increase in specific surface area would increase the bonding area between the particles, thereby enhancing the TS of tablet.

Different from dry granulation, the change of tabletability in wet granulation has not been fully investigated. Various wet granulation techniques, such as high shear wet granulation (HSWG), fluidized bed wet granulation (FBD) and twin screw wet granulation (TSWG), as well as the complex interactions between formulation materials and process variables [31], increased the research difficulty. The phenomenon of severe loss of tabletability was also termed as over-granulation in wet granulation, and it often happened on plastic materials like microcrystalline cellulose (MCC) [32]. The possible mechanisms were first revealed by Badawy et al. [33] that the particle porosity was reduced. Later, Shi et al. [34] proved that the MCC granule tabletability was decreased with increasing granule size, and granule size enlargement was the key mechanism for over-granulation in HSWG. In another case, Khorsheed et al. [35] conducted TSWG with microcrystalline cellulose, mannitol, and dibasic calcium phosphate anhydrous as the research objects. The loss of tabletability was observed for microcrystalline cellulose and mannitol 100 SD, and it was caused by increased granule strength but was independent on the granule size. By contrast, the brittle materials mannitol C160 and dibasic calcium phosphate anhydrous maintained their tabletability due to the reduced or constant granule size of materials after TSWG. Osei-Yeboah et al. [36] showed that a combination usage of brittle and plastic excipients could solve the problem of tabletability reduction. Except for the investigations on common fillers, such as microcrystalline or lactose, some unusual mechanisms of change of tabletability were found. For instance, the polymorphic transition from delta- to beta-mannitol occurred during TSWG, which improved the tabletability of delta-mannitol [37]. Another recent example found that the breakage of lengthy crystals of glucose during TSWG significantly increased the tabletability of glucose [38]. It could be seen that the reported literature on the reduced tabletability of materials in wet granulation was important for designing a robust WG process. However, these experiences were limited to single materials or particular formulations and a more comprehensive understanding is needed for identifying effective solutions to the loss of tabletability problem in WG process.

Recently, several reports have adopted the concept of material library, which involved building of material database for relevant pharmaceutical materials and understanding how material properties influence the process behaviors and the critical quality attributes of finished dosage forms [39]. The application scenarios of material library in the pharmaceutical formulation and process development mainly include: (1) supporting the identification of equivalent or surrogate materials with similar properties to expensive, scarce, or toxic active pharmaceutical ingredients (APIs) [40]; (2) facilitating the selection of materials with maximal variability to enlarge the design space [41], or reducing the number of relevant characterization methods in order to accelerate drug development [42]. (3) Establishing material classification systems that enable the risk assessment of any new material. For instance, Dai et al. [43] proposed a compression behavior classification system with the help of 9 compression parameters of 70 directly compressed pharmaceutical materials, and it was found that two categories of powders possessed unacceptable tabletability over a wide pressure range. (4) Modeling the relationships among raw material attributes, process parameters, and critical quality attributes of a product to support the formulation and process optimization. This is of great significance to achieve the science-based pharmaceutical development within the framework of quality by design (QbD) [44]. With regard to tablet design, the material library approach has been mainly applied to the direct compression process. For instance, Hayashi et al. [45] built a material library consisting of 81 kinds of API, the machine learning algorithms were used to model the relationships between input variables (material properties and compression pressure) and output variables (tablet tensile strength and disintegration time). Besides, the material library approach has been used to understand the impacts of material properties on process performance of unit operations, such as roll compaction [23], loss-in-weight feeding [46], etc. As far as we know, there is no research on the application of the material library approach in understanding the change of tabletability in wet granulation and tableting processes.

HSWG is the most popular granulation technique in the tableting process since it has the advantages of efficient mixing of the powder blend, short processing time, low liquid amount, and short drying time compared with other granulation methods [47]. Most importantly, HSWG is suitable to produce high drug loading formulations, such as metformin hydrochloride tablets [48], metronidazole tablets [49], and acetaminophen tablets [50], etc. In this paper, the material library method was used to identify the changes of tabletability of different materials during the tablet manufacturing by HSWG. The rest of the paper was organized as follows. Section 2 describes the details of the material library, the experimental design for high shear wet granulation and tableting (HSWGT) and the material characterization methods for raw materials, intermediates, and tablet products. The calculation equations for reworking potential as well as a new index (i.e., relative change of tabletability, *CoT*_r_) for evaluating the change of tabletability are given. In Section 3, the physical properties and compression behaviors of powdered materials and granules are first compared from both the univariate and multivariate aspects. Then, the phenomenon of tabletability change is analyzed both qualitatively and quantitatively. The advantages of the *CoT*_r_ index over the reworking potential are discussed. With the help of *CoT*_r_, a tabletability change classification system (TCCS) is developed. Moreover, the differences in requirements of critical material attributes for DC and HSWGT were identified. Section 4 concludes and brings froward future research suggestions. 

## 2. Materials and Methods

### 2.1. Materials

To establish the material library 30 materials including 18 pharmaceutical excipients and 12 natural product powders (NPPs) were used. All pharmaceutical excipients were purchased commercially, and these excipients played the roles of diluents, binders, and disintegrants in tablet formulations. The selected excipients were suitable for direct compression and/or granulation processes. According to the deformation characteristics of materials, the excipients could be divided into three types, i.e., the plastic, the brittle, and the elastic. Microcrystalline cellulose PH101 (MCC PH101) and silicified microcrystalline cellulose (SMCC) were typical plastic excipients [51]. The brittle excipients included mannitol, calcium phosphate (CaP), dibasic calcium phosphate (DCP), dibasic calcium phosphate anhydrous (DCPA), lactose granulac 200 (Lac G200), and lactose flowlac 100 (Lac F100) [52,53,54]. The elastic excipients contained pregelatinized starch (PGS), soluble starch, and dextrin [55,56,57]. Among these excipients, SMCC and lactose cellactose 80 (Lac C80) were co-processed excipients. All NPPs were provided by the Beijing Tcmages Pharmaceutical Co., Ltd. (Beijing, China) and were prepared from 12 medicinal plant that involved commonly used medicinal parts, e.g., roots, rhizomes, bark, fruits, flowers, and aerial parts. The manufacturing processes of each NPP included a series of unit operations, such as pretreatment, extraction, filtration, concentration, and spray drying. Compared to excipients, NPPs had the characteristics of multiple compositions, high hygroscopicity, low glass transition temperatures, poor flowability, and compactability [58]. NPPs were chosen in expectation of enriching the diversity of the material library. The name, lot number, and supplier for each material are provided in Table S1 in Supplementary Materials S1.

### 2.2. Characterization of Powders

The pharmaceutical excipients were sifted using a sieve with an aperture size of 850 μm to remove any lumps. Then, the sieved excipient powders were spread on a tray and were placed in a blast drying oven set at 60 °C to dry for 2 days [59]. The dried powders were then put into a ziplock bag and equilibrated in an environment of relative humidity (RH) maintained at 50% and temperature of 25.0 ± 2.0 °C for at least three days. The NPPs were sensitive to heat and humidity, so they were only sifted by an 850-μm sieve to obtain powders with homogeneous size before use.

All powdered materials were characterized by 19 physical parameters. Among them, 12 parameters were measured or calculated by standard testing procedures of the SeDeM expert system methodology [60,61], and they were bulk density (*D*_a_, g·mL^−1^), tapped density (*D*_c_, g·mL^−1^), inter-particle porosity (*Ie*), Carr’s index (*IC*), Hausner ratio (*IH*), angle of repose (*AOR*, °), flow time (*t″*, min), cohesion index (*Icd*, N), loss on drying (*%HR*), hygroscopicity (*%H*), particle size less than 50 μm (*%Pf*), and homogeneity index (*Iθ*). The dimensions of powders could be expressed by *D*_a_ and *D*_c_. The parameters *IC*, *Ie* and *Icd* characterized the compressibility of powders. The descriptors *AOR*, *t″*, and *IH* reflected the flowability of powder. The stability of the powder could be described by the parameters *%HR* and *%H*. The physical properties *%pf* and *Iθ* represented the uniformity of powder. Other than the SeDeM parameters, the remaining seven physical properties were true density (*D*_t_, g·cm^−3^), particle sizes (i.e., *D*_10_, *D*_50_ and *D*_90_), particle size distribution (*span*), solid fraction (*SF*_p_), and porosity (*ε*_p_), all of which were determined by established methods [62]. The testing results of material properties of 30 materials in the material library are achieved in a homemade database named intelligent TCM (iTCM), and all data are shown in Supplementary Materials S2.

### 2.3. High Shear Wet Granulation Experiment

#### 2.3.1. Experiment Design

As a fact, multiple process factors of HSWG and their interaction could result in complex experiment programs even for simple formulations or single materials. The purpose of the experimental design here was to successfully produce acceptable granules for each material in the material library, but not to fine tune the process parameters. The arrangement of the experiment design is shown in Table 1. The main process parameters considered were the type of wetting agent, the liquid to solid (L/S) ratio, the impeller speed, and the addition rate of wetting agent. The design principles of the four parameters were as follows.

In order to avoid the introduction of additional substance, deionized water and 95% (*v*/*v*) ethanol were chosen as wetting agents because of their volatile properties. NPPs and five starch derivatives (i.e., cold water-soluble starch, cold water-insoluble starch, dextrin, maltodextrin, and pregelatinized starch) were not easy to wet uniformly, and a sticky wet mass or hard granules could be produced when using water as the wetting agent. For those materials, 95% (*v*/*v*) ethanol was used as the wetting agent. Except for the five starch derivatives, the remaining 13 excipients applied water as the wetting agent to induce their viscosity in forming granules. The L/S ratio was determined for each material based on the results of preliminary experiments (details not shown). The principle of determining the L/S ratio was to avoid the slurry state or formation of large agglomerates. 

The impeller speed and the addition rate of wetting agent in this study were referred to literatures with the same or similar granulator scale [63,64]. NPPs have smaller particle size, stronger inter-particle cohesion, and poorer flowability compared to excipients. Therefore, a high impeller speed of 600 rpm was set in the wet granulation of NPPs, which allowed the powders to flow freely and contact sufficiently with the wetting agent. Meanwhile, a low impeller speed of 300 rpm was applied for the wet granulation of pharmaceutical excipients. The chopper speed was kept at 1600 rpm for all experiments. The addition rate of the wetting agent was designed to match the impeller speed. When ethanol was used as the wetting agent, the high-level combination of the impeller speed and the addition rate was used to achieve rapid granulation and to avoid the volatilization of ethanol. In all experiments, the addition time of wetting agent ranged from 1.2 to 15.6 min depending on the L/S ratio used.

#### 2.3.2. Process Description

An approximate 300 g powders were poured into a laboratory scale high shear granulator with a 2 L bowl (SHK-4, Xi’an Runtian Pharmaceutical Machinery Co., Ltd., Xi’an, China). Then, the dry mixing was performed for 1 min at the impeller speed of 300 rpm and without working of the chopper. After dry mixing, the wetting agent was added into the granulation bowl using a peristaltic pump (BT00-100 M, Baoding Longer Precision Pump Co., Ltd., Baoding, China) at defined impeller and chopper speeds. The wet massing time was lasted for 180 s. At the end of the granulation process, the wet mass was sifted by a 10-mesh standard sieve and the wet granules were spread out on a tray and transferred into an oven for drying at 55 °C. The drying time was controlled until the moisture content of dried granules was close to that of corresponding powders. Dry granules were separated by using a vibration screen with two standard sieves (ZNS-300, Beijing Kingslh Technology Development Co. Ltd., Beijing, China). After sieving, the granules with size fraction of 125–250 μm were used for the subsequent tableting process [65,66,67]. The purpose of selecting the same particle size fraction is to facilitate the comparison of the physical and mechanical properties of granules made from different materials.

### 2.4. Tableting Process

The pretreated powders and the prepared granules were compacted respectively into tablets by using a single punch tablet press machine (C&C600A, Beijing C&C CAMBCAVI Co., Ltd., Beijing, China) which was equipped with the flat faced punch and die with 10 mm in diameter. The magnesium stearate was used to lubricate the punch surfaces and the die walls before each compaction. After lubrication, the powders or granules were manually filled into the die and were compacted. Considering the different bulk densities of the materials, the filling mass of the material was set to 300 mg or 350 mg to ensure the smooth ejection of the tablet. For each material, powdered or granulated, six compaction pressures from 10 MPa to 140 MPa (1 kN = 12.74 MPa) were applied to obtain tablets with different hardness. At least two tablets were obtained under each pressure. The tableting speed was maintained at 25 tablets per minute. The prepared tablets were sealed in a ziplock bag. After being stored for 24 h, the weight, diameter, thickness, and diametrical crush force of tablets were measured. The tablet weight was acquired by using an analytical balance (GL124-1SCN, Beijing Sanfu Hezhong Technology Development Co., Ltd., Beijing, China). The diameter and thickness of tablets were measured with a digital calliper (547–401 Digimatic Caliper, Mitutoyo, Japan). The diametrical crush force of tablets was recorded by a tablet hardness tester (YPD-500, Shanghai Huanghai medicine inspection instrument Co., Ltd., Shanghai, China). The tensile strength (TS) and the solid fraction (SF) of tablets are calculated by Equations (1) and (2), respectively.

(1) $$TS=\frac{2F}{\Pi DH}$$

where *F* (N) is the tablet crush force, *D* (mm) is the tablet diameter, and *H* (mm) is the tablet thickness [68].

*SF* = 1 − *ε* (2)

(3) $$\varepsilon =\frac{\rho_{app}}{\rho_{true}}\;$$

(4) $$\rho_{app}\;=\frac{m}{\Pi \frac{D2}{4}H\;}\;$$

where *ε* is the tablet porosity, *ρ*_app_ is the apparent tablet porosity, *ρ*_t_ (g·cm^−3^) is the true tablet density, and *m* (g) is the tablet weight. *ρ*_t_ (g·cm^−3^) is equal to the true density of the material.

### 2.5. Compression Models

After the tableting and tablet characterization experiments in Section 2.4, the compression curves interpreting respectively the compressibility (i.e., pressure vs. porosity), compactability (i.e., porosity vs. TS), and tabletability (i.e., TS vs. pressure) properties of a material could be plotted. Then, different compression models were used to fit the compression curves under specified pressure ranges in order to acquire the CBCS parameters. When using the Shapiro model, the pressure range was 0–50 MPa. For other compression models in the CBCS method, the pressure range was 10–140 MPa.

#### 2.5.1. The Kawakita Model

The Kawakita equation [69] was developed to study powder densification using the degree of reduction in volume, *C*, and is expressed as Equation (5):

(5) $$C=\frac{V_{0}-V_{p}}{V_{0}}\;\;\frac{abP}{1+bP}\;$$

where *a* and *b* are Kawakita compression parameters which can be determined by linear regression using the linearized form as Equation (6)

(6) $$\frac{P}{C}=\;\;\frac{P}{a}+\;\;\frac{1}{ab}$$

where *V*_0_ is the initial in-die volume of the powder, and *V_P_* is the powder volume after the application of pressure *P*. *V*_0_ is set from the bulk density transformed into a corresponding height in die. *a* and *b* are constants which can be obtained from the slope and intercept of the *P/C* vs. *P* plot, respectively. The parameter *a* represents the maximal strain or degree of compression at maximal pressure (*C*_max_). The parameter *b*^−1^ describes the pressure to reach *a*/2, which was correlated to the plasticity (yield pressure, Heckel) and can be seen more simply as deformation capacity.

#### 2.5.2. The Shapiro Model

As a descriptor of the powder compression process in region I of the *SKH* (Shapiro–Konopicky–Heckel) compression profile, the Shapiro compression parameter *f* was derived from the Shapiro general compaction equation (GCE) [70].

*Ln (ε) = Ln (ε_0_) − kP − fP*^0.5^   (7)

where *ε* is the porosity of the powder bed, *ε*_0_ is the initial porosity of the powder bed corresponding to zero pressure and is obtained by dividing the measured bulk density and true density, *P* is the applied compression pressure, and *k* and *f* are constants.

#### 2.5.3. The Heckel Model

The Heckel equation [71,72] describes the relationship between the logarithm of the inverse of the porosity and the applied compression pressure, *P*. The Heckel equation was derived based on the assumption that the in-die densification of the bulk powder obeys the first order kinetics as:

(8) $$Ln\;\frac{1}{\varepsilon }=kP+A$$

where *ε* is the porosity of the powder bed, *P* is the applied compression pressure, and *k* and *A* are constants. The inverse of *k* is the mean yield pressure, which represents the limit of plastic deformation of materials or the resistance of a material to deformation [73].

#### 2.5.4. The Gurnham Model

Considering several drawbacks of the Heckel equation, the Gurnham equation was proposed to characterize the deformation behavior of pharmaceutical material [74]. It is used to describe the relationship between compression pressure and compact porosity:

(9) $$\varepsilon =-\frac{1}{K}\;\;ln(\frac{P}{P_{0}})$$

where *ε* is the porosity of the tablet, *P* is the applied compression pressure, *P*_0_ is the pressure required to produce a zero-porosity compact, and *K* is related to the compressibility resistance of the powder.

#### 2.5.5. The Ryshkewitch-Duckworth Model

The Ryshkewitch–Duckworth (R-D) equation can be used to describe the logarithmic relationship between the tensile strength and porosity of a compact [75,76]:

(10) $$Ln(\frac{TS}{TS_{0}})=-\;k_{b}\times \varepsilon \;$$

where *ε* is the porosity of the compact, *TS*_0_ is the tensile strength of the same material at zero porous, and *k_b_* is a constant representing the bonding capacity of material particles. A higher value of *k_b_* corresponds to the weaker bonding of primary particles [77].

#### 2.5.6. The Power Model

Previous studies have demonstrated that a simple power function could describe the relationship between the tablet tensile strength and the tableting pressure [43].

*TS = dP^g^*  (11)

where *TS* is the tensile strength of the tablet and *P* is the compression pressure. The parameters *d* and *g* are constants, which were expressed as the tabletability and pressure sensitivity of materials, respectively.

### 2.6. Evaluation of Change in Tabletability

#### 2.6.1. The Reworking Potential

**Figure 1:**
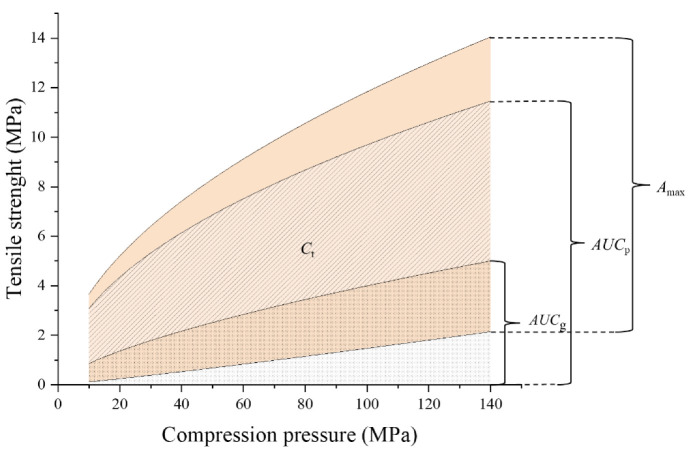
The schematic diagram for evaluation of the tabletability change.

The reworking potential (*RP*) index was first proposed by Malkowska et al. [8]. In this method, the tabletability change of the material was calculated by dividing the area under the tensile strength vs. compression pressure profile of granules (*AUC*_g_) to the area under the tensile strength vs. compression pressure profile of powders (*AUC*_p_), as shown in Figure 1. The calculation equation is as follows:

(12) $$\mathrm{Reworking}\;\mathrm{potential}=\frac{AUC_{g}}{AUC_{p}}\;\times \;100\%\;$$

#### 2.6.2. The Relative Change of Tabletability

In order to compare the change of tabletability of different materials based on the concept of material library, this paper proposed a new index, namely relative change of tabletability (*C_O_T*_r_). First, the tensile strength vs. pressure curves for all powders in the material library and granules prepared from the powders were plotted in order to determine the maximum area under the curve (*AUC*_max_) and minimum area under the curve (*AUC*_min_). The maximum change of tabletability (*A*_max_) is defined as the difference between *AUC*_max_ and *AUC*_min_, as shown in Figure 1. Then, for a particular material, the change of tabletability (*C*_t_) is calculated as the difference between the area under the tabletability curves of the powder (*AUC*_p_) and the area under the tabletability curves of the granule (*AUC*_g_). Finally, the relative changes of tabletability (*C_O_T*_r_) of a material could be expressed as dividing *C*_t_ by *A*_max_ as follows:

(13) $${CoT}_{r}=\frac{C_{t}}{A_{\max}}\;\;\frac{AUC_{g}-AUC_{p}}{AUC_{\max}-AUC_{\min}}\times 100\%\;$$

### 2.7. Multivariate Data Analysis

Principal component analysis (PCA) is performed to reveal latent structures in the data set and to identify the grouping tendency of materials. The PCA uses a vector space transformation to reduce the dimensionality of the available data set [78]. The partial least squares (PLS) regression method is used to model the relationship between the input data matrix and the output data matrix. Performing a PLS analysis can help reduce the manifest variables to a few latent variables that are linear combinations of the original variables. Before modeling, data need to be centered and scaled to eliminate dimensional differences [79]. The quality of a PLS model can be evaluated by the R2Y value, i.e., the correlation between the observed and predicted values for the studied response, and the Q2 value, i.e., the correlation between the observed and cross-validated predicted response. The higher the R2Y and Q2 value are, the better the response can be explained and predicted. The PCA and PLS algorithms were executed using the SIMCA 13.0 (Umetrics, Umea, Sweden) software.

## 3. Results and Discussions

### 3.1. Comparison of Physical Properties of Powders and Granules

According to the experimental program in Section 2.3, each material in the material library was granulated by high shear wet granulation under designed conditions. After the wet granules were dried and sieved, 30 kinds of granular materials were prepared, and their physical properties were characterized according to the procedures in Section 2.2. For each kind of granule, 19 physical parameters were obtained, and the results are presented in Supplementary Materials S2. The differences in physical properties between powders and granules were analyzed from both univariate and multivariate perspectives.

#### 3.1.1. Univariate Analysis

**Figure 2:**
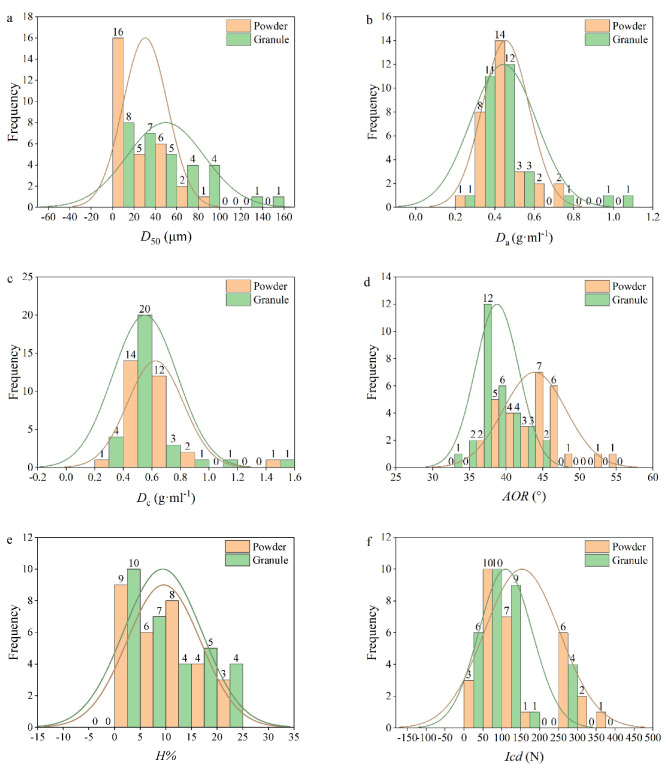
The histograms of physical properties for powders and granules. (**a**) the median diameter *D*_50_. (**b**) the bulk density *D*_a_. (**c**) the tapped density *D*_c_. (**d**) the angle of repose *AOR*. (**e**) the hygroscopicity *%H*. (**f**) the cohesion index *Icd*.

Six physical parameters were chosen to perform the single variable comparison. The histograms and corresponding Gaussian fits of every physical property of powders and granules are shown in Figure 2.

The *D*_50_ is the medium value of the particle size distribution, which is one of the important parameters to characterize the particle size. The histograms of *D*_50_ for both powders and granules are illustrated in Figure 2a. The *D*_50_ values of ungranulated powders varied from 4.67 μm (CaP, No. E13) to 81.13 μm (SMCC, No. E14). Moreover, the *D*_50_ values of granules ranged between 7.53 μm (CaP, No. E’13) and 152.83 μm (DCP, No. E’10). It could be found that the particle size of CaP did not increase significantly after HSWG, indicating that the particles of this material could not be bonded effectively by using water as the granulation agent during wet granulation. This phenomenon also confirmed that the effects of granulating agent types and granulation parameters on the tabletability of different materials deserve to be further studied. By contrast, the material with the largest change of particle size was DCP, whose *D*_50_ was increased from 49.56 μm to 152.83 μm after granulation. Conversely, the *D*_50_ of mannitol (No. E11) was reduced from 71.80 μm to 16.53 μm after HSWG. The dissolved mannitol may recrystallize into smaller crystals during the granulation process [35], which gave granules smaller particle sizes and higher surface area than primary powders. In Figure 2a, the mean value of *D*_50_ of powders is 30.43 μm, which is smaller than that of granules, 49.46 μm. The distribution width of *D*_50_ values of all granules is larger than that of all powders mainly due to the different number of materials distributed in the range of *D*_50_ greater than 70 μm. To be more specific, there are 10 granular materials with *D*_50_ greater than 70 μm, whereas only one powdered material is included in the same range. Overall, after HSWG, the particle sizes of most materials would be increased since the wetting agent could induce the self-adhesion of materials to form large agglomerates. 

Figure 2b depicts the frequency distributions of bulk densities for both powders and granules. The range of bulk densities of ungranulated powders was 0.26–0.79 g·mL^−1^. The Cinnamomi Cortex (CC, No. Z11) had the smallest *D*_a_ value, and the DCPA (No. E8) had the largest *D*_a_ value. Although similar particle sizes were observed for powders Z11 and E8, they had different inter-particle porosities. Specifically, the DCPA had a small inter-particle porosity (*Ie* = 0.57), while the Cinnamomi Cortex had a large inter-particle porosity (*Ie* = 1.28). The bulk densities of granules varied from 0.26 g·mL^−1^ (Polyvinylpolypyrrolidone (PVPP), No. E’16) to 1.00 g·mL^−1^ (DCPA, No. E’8). After granulation, the bulk densities of 11 materials were increased, and the bulk densities of 16 materials were decreased. The bulk densities of Sophorae Flavescentis Radix (SFR, No. Z10), SMCC (No. E14) and MCC (No. E15) remained unchanged. According to the MCS working group [22], the desired bulk density of materials for tableting should be no less than 0.3 g·mL^−1^. By that measure, the powder of Cinnamomi Cortex and the granule of PVPP had the potential risk of die filling during the tableting process. The mean values of *D*_a_ of all powders and granules were 0.45 g·mL^−1^ and 0.44 g·mL^−1^, respectively. In addition, it could be seen that the distribution range of bulk densities of granules was slightly wider than that of powders due to the large bulk density of granules of DCPA (1.00 g·mL^−1^) and DCP (0.93 g·mL^−1^). Generally, it could be concluded that the bulk density of materials did not change significantly after HSWG, except for the two DCP excipients.

The histograms of tapped densities for both powders and granules are shown in Figure 2c. Tapping and vibration caused rearrangement of the particles, reducing the void space and increasing the granule density. The *D*_c_ values of ungranulated powders ranged from 0.39 g·mL^−1^ (CC, No. Z11) to 1.44 g·mL^−1^ (DCPA, No. E8). The *D*_c_ values of granules varied between 0.31 g·mL^−1^ (PVPP, No. E’16) to 1.43 g·mL^−1^ (DCPA, No. E’8). It could be seen that the HSWG process would not affect the *D*_c_ of DCPA. It is worth noting that the *D*_c_ of croscarmellose sodium (CMC-Na, No. E18) had the largest difference before and after granulation, from 0.73 g·mL^−1^ to 0.45 g·mL^−1^. This was attributed to the swelling of CMC-Na in contact with water [80,81], resulting in the increased particle porosity *ε*_p_, which changed from 0.69 to 0.75. The mean value of *D*_c_ of all granules (0.55 g·mL^−1^) was smaller than that of powders (0.62 g·mL^−1^). This indicated that the particle size enlargement had a negative effect on the rearrangement of materials. [82]. 

The angle of repose reflects the internal friction and cohesion properties of the bulk material. The larger the angle of repose was, the larger the friction coefficient and the worse the flowability of the material was. The *AOR* values of ungranulated powders ranged from 36.3° to 54.5°. The minimum and the maximum *AOR* values could be found in CaP (No. E13) and Lac G200 (No. E7), respectively. Besides, the *AOR* of DCPA powder was 52.8° second only to Lac G200. The Lac G200 and DCPA were reported to possess the rough particle morphology and irregular particles, resulting in their poor flowability [18,83]. The *AOR* values of granules were distributed between 33.2° (Mume Fructus (MF), No. Z’6) and 45.8° (DCPA, No. E’8). It was generally accepted that when the *AOR* was less than 41°, the material could meet the requirements for flowability in the tableting process [84]. After granulation, the *AOR* values of 25 kinds of granules were smaller than 41°. The mean value of the *AOR* of all granules (38.8°) was smaller than that of all powders (43.9°). For instance, the *AOR* of granules of Lac G200 was 36.8°, which demonstrated that the flowability of this material was well improved after wet granulation. Besides, the kurtosis of the *AOR* Gaussian curve of powders was smaller than that of the granules. As can be seen from Figure 2d, 21 granular materials are grouped in the range *AOR* of 35°–40°, while only seven powders are included in this range. From the above analysis, it could be confirmed that wet granulation had the advantage of improving the flowability of materials.

Figure 2e displays the comparison of hygroscopicity for powders and granules. Generally, materials with strong hygroscopicity would be accompanied by strong cohesion and poor flowability [85]. The distribution range of the hygroscopicity of ungranulated powders was 2.92 × 10^−3^%–23.2%. The Lac F100 (No. E6) hardly absorbed any water and it had the smallest value of hygroscopicity, whereas the Menthae Haplocalycis Herba (MHH, No. Z4) had the largest value of hygroscopicity, due to its small particle size (*D*_50_ = 9.48 μm), large specific surface area, and high powder porosity (*ε*_p_ = 0.77). The hygroscopicity of all granules varied between 0.03% (DCPA, No. E’8) to 23.9% (Chuanxiong Rhizoma (CxR), No. Z’7). Generally, the pharmaceutical excipients had weak hygroscopicity, whereas NPPs had strong hygroscopicity [58]. This phenomenon was also applicable to granular materials, since the ranges of hygroscopicity for all granular excipients and all granular NPPs were 0.03–16.5% and 9.38–23.9%, respectively. The mean values of hygroscopicity for powders and granules were 9.60% and 9.38%, respectively. In addition, the distribution width of hygroscopicity of all granules was equivalent to that of powders. This indicated that the hygroscopicity of materials did not change much after granulation.

The cohesion index is closely related with the material’s compressibility [86]. The smallest *Icd* value of ungranulated powders was 35.9 N (PGS, No. E5). The MCC PH101 (No. E15) had the largest value of *Icd* (i.e., 384 N). The *Icd* values of granules changed between 20.9 N (PGS, No. E’5) and 280 N (Polygoni Multiflori Radix Praeparata (PMRP), No. Z’1). The pregelatinized starch maintained the low level of compressibility before and after granulation, which might be attributed to its dense texture and small porosity for both the powders (*ε*_p_ = 0.59) and granules (*ε*_p_ = 0.53). The mean value of the *Icd* of all powders (153 N) was larger than that of all granules (110 N), meaning that materials were prone to reduce compressibility after HSWG. However, after a carefully looking at Figure 2f, the change of mean value is mainly caused by the number of materials with *Icd* values larger than 200 N. As shown in Figure 2f, there are nine powdered materials with *Icd* values larger than 200 N. Nevertheless, only four kinds of granules have *Icd* values larger than 200 N. Besides, there were some special cases. For example, Mume fructus had a larger *Icd* value (259 N) after granulation than its powder form (*Icd* = 78.5 N). This suggested that materials with increased *Icd* values gained better compressibility after HSWG. The diversification of changes of *Icd* values was considered to be beneficial to the study of the change of tabletability.

#### 3.1.2. Principal Component Analysis

**Figure 3:**
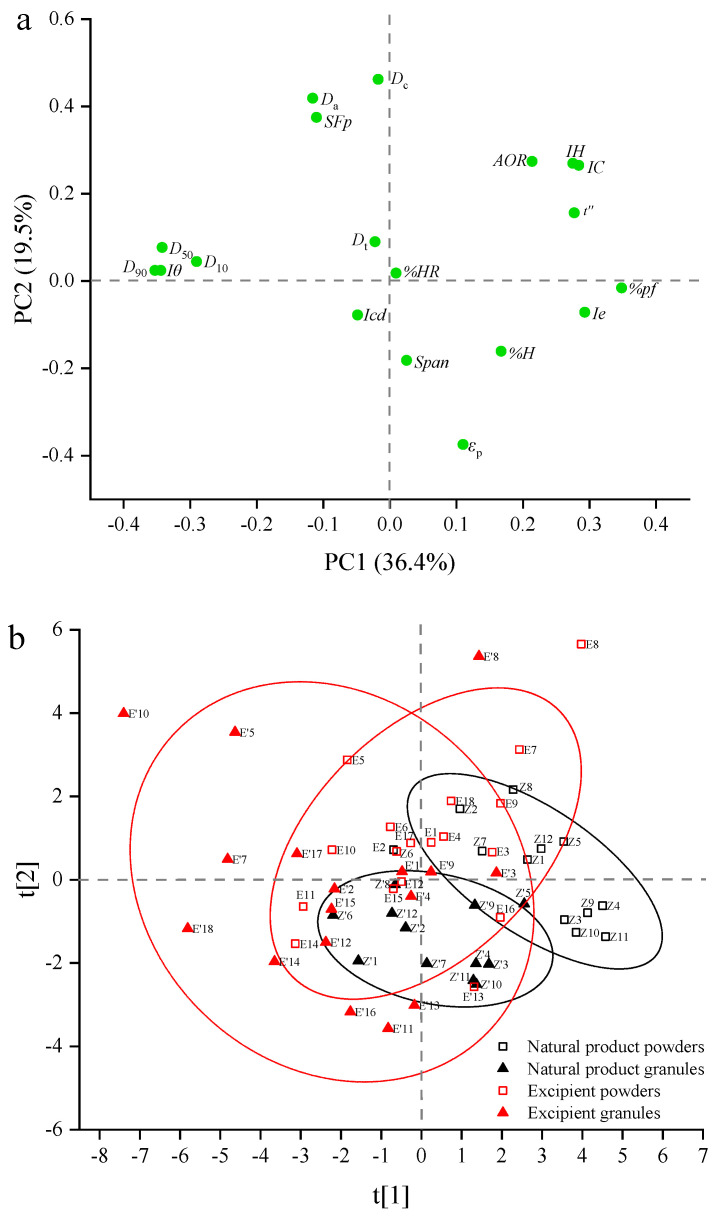
The PCA analysis for the physical properties data base on the first two principal components. (**a**) the loading plot; (**b**) the score plot. (The black squares represent NPPs. The black triangles represent NPGs. The red squares represent excipient powders. The red triangles represent excipient granules. Two black circles are 95% confidence ellipses for NPPs and NPGs, respectively. Two red circles are 95% confidence ellipses for excipient powders and excipient granules, respectively).

The principal component analysis was performed with the material properties data matrix (size 60 × 19) consisting of 60 samples (30 ungranulated powders and 30 granules). 19 physical properties were used as matrix variables. The data matrix was pre-processed by mean-centering and scaling to unit variance before PCA analysis. In general, PCA decomposed a data matrix in such a way that the first principal component (PC1) represented the direction with the largest variation in the data, and PC2 was oriented orthogonally to PC1 and reflected the second largest source of variation in the data. As shown in Figure 3, the first three principal components were able to summarize 70.9% (PC1, PC2 and PC3 accounted for 36.4%, 19.5% and 15%, respectively) of the total variation in the original dataset. Figure 3a was the loading plot of the data matrix. The objects on the same side of a PC were positively correlated, and the opposite ones were negatively correlated. Objects close to each other or clustered in groups had similar features, while objects that were situated far away from each other were dissimilar [87]. The physical properties of materials associated with PC1 were mainly the particle size parameters (i.e., *D*_10_, *D*_50_, *D*_90_, *Iθ* and *%pf*), the flowability parameter *t″* and the compressibility parameters *Ie*. The particle size parameters (i.e., *D*_10_, *D*_50_, *D*_90_ and *Iθ*) were located on the negative side of PC1 and were opposite to the flowability and compressibility parameters. This result implied that materials with larger particle sizes were prone to have better flowability but worse compressibility. The properties contributing to PC2 were mainly the density parameters (i.e., *D*_c_, *D*_a_, *SF*_p_ and *ε*_p_) and the particle size distribution parameter *span*.

Figure 3b shows the score plot of the data matrix. The NPPs, the natural product granules (NPGs), the excipient powders, and the excipients granules are depicted as a black square, black triangle, red square, and red triangle in Figure 3b, respectively. Two 95% confidence ellipses in the black were used to represent the distribution space of physical properties for the NPPs and NPGs, respectively. The distribution space of physical properties for the excipient powders and the excipient granules were characterized by two red 95% confidence ellipses. It could be found that NPPs were mainly distributed on the positive axis of PC1, and excipients were mainly situated on the negative axis of PC1, indicating that excipients generally had larger particle sizes, better flowability but poorer compressibility than NPPs. In addition, it could be seen that there was almost non-overlapping of the 95% confidence ellipses for NPPs and NPGs, and this was mainly due to the significant increase in particle sizes and decrease in angle of repose. However, the 95% confidence ellipses of the excipient powders and the excipient granules had a large overlapping area, showing that some excipients had no obvious changes in physical properties after granulation. Compared with the ungranulated powders, most of the granulated materials tended to move to the lower left of the score plot, suggesting the granulation had the capabilities of increasing the particle sizes, improving the flowability and decreasing the inter-particle porosity. There were also some cases contrary to the general trend. For example, the point representing the granule of mannitol (No. E11) was located on the lower right of the score plot compared with the point of the mannitol powders, due to the decrease in particle sizes after granulation. This was consistent with the univariate analysis of particle size changes.

### 3.2. Comparison of Compression Behavior of Powders and Granules

#### 3.2.1. Compression Model Fitting Results

Each material, in the form of ungranulated powders or granules, was compressed into tablets with different hardness under the pressure range of 10–140 MPa according to the procedures in Section 2.4. The curves of porosity–pressure, porosity–*TS,* and *TS–*pressure that respectively reflected the compressibility, compactability, and tabletability of materials could then be plotted. After that, CBCS parameters could be obtained by using different compression models in Section 2.5 to fit the curves. Especially, for CaP (No. E13), whether in the form of powders or granules, the obtained tablets would undergo capping when the applied pressure exceeded 70 MPa. Therefore, this material was not included in the following comparative study of compression behaviors. The goodness of fit of each compression model was evaluated by the determinant coefficient R^2^ and the root mean square error (RMSE). The model fitting results as well as the CBCS parameters for all materials are shown in Supplementary Materials S2. In general, the R^2^ values of 339 out of 348 compression models were higher than 0.9, demonstrating the good fitness. The R^2^ values of remaining nine compression models were higher than 0.8, revealing the favorite fitness. There are mainly two possible reasons to which the inferior fitting results of the nine compression models can be attributed. The first reason was that the *TS* vs. compression pressure curves of materials, such as β-CD and CWSS, were approximately straight lines parallel to the X-axis when the compression pressure was greater than 60 MPa. Therefore, they could not be fitted well with the Power model. The second reason was that the data points on the *TS* vs. compression pressure curves for materials, e.g., CWIS granules, dextrin granules, and pregelatinized starch granules, were relatively scattered. It was inferred that granules made from starch derivatives were sensitive to the die filling process. 

For the sake of comparison, the lowest and highest values of each CBCS parameter are shown in Table 2. The parameter *a* in Kawakita represented the maximal engineering strain of the raw powders or granules. The larger the value of *a* was, the easier the powder bed of the materials could be compressed. The Kawakita parameter *a* of ungranulated powder varied from 0.708 (DCP, No. E10) to 1.05 (CWSS, No. E2), and the descriptor *a* of granules changed between 0.746 (DCPA, No. E’8) and 1.05 (CWIS, No. E’1). This indicated that soluble starch was easier to compress than DCP excipients, which was related to the porous structure of soluble starch and the high density of DCP excipients [88,89]. The parameter *ab* in the Kawakita model could be used as an indication of the incidence of particle rearrangement during powder compression and a high *ab* value corresponded to high degree of particle rearrangement [90]. The values of rearrangement index *ab* of ungranulated powders ranged from 6.58 × 10^−2^ (PMRP, No. Z1) to 0.283 (Lac G200, No. E7). The *ab* values of granules varied from 3.86 × 10^−2^ (CMC-Na, No. E’18) to 0.288 (β-cyclodextrin, β-CD, No. E’9), suggesting that CMC-Na granules and PMRP powders had nearly no initial particle rearrangement. 

The parameter *f* was used to evaluate the degree of particle fragmentation during the initial compression process. A material with a large *f* value was prone to particle fragmentation. The Shapiro parameter *f* of ungranulated powders changed between 8.84 × 10^−2^ (CWSS, No. E2) and 0.362 (Lac G200, No. E7), and the *f* values of granules ranged from 3.12 × 10^−2^ (PGS, No. E’5) to 0.326 (Mannitol, No. E’11). This showed that the two starch excipients had a limited inclination of particle fragmentation. Nevertheless, the lactose and mannitol excipients were easily fragmented during the initial compression process. 

The Heckel mean yield pressure, *P*_y_, signified the plastic deformation capacity of powders. The smaller the *P*_y_ was, the better plastic deformation ability of material was achieved. The distribution range of the Heckel parameter *P*_y_ for powders was between 41.6 (MHH, No. Z4) and 686 (DCP, No. E10). The *P*_y_ value of granules varied from 38.9 (CWIS, No. E’1) to 684 (DCPA, No. E’8). This demonstrated that the DCP excipients had poor plastic deformation ability, while the starch excipients had good plastic deformation ability. This could be attributed to the high densification degree of DCP and the low densification degree of starch. 

The parameter *K* in the Gurnham equation was related to the compressibility resistance of the powders and granules. The larger the value, the stronger the compression resistance of the material was obtained, and the more difficult for it to be compressed. The maximum and minimum values of the Gurnham parameter *K* of the powders were 6.35 (Buddlejae Flos (BF), No. Z12) and 24.5 (DCPA, No. E8), respectively. The largest and the smallest values of the parameter *K* of granules were 5.87 (CMC-Na, No. E’18) and 24.9 (DCPA, No. E’8). 

The parameter *k*_b_ in the Ryshkewitch–Duckworth equation represented the bonding capacity of powders and granules. The larger the *k*_b_ value was, the worse bonding capacity of materials was acquired. The values of the Ryshkewitch–Duckworth parameter *k*_b_ of powders were distributed between 4.54 (β-CD, No. E9) and 22.8 (DCPA, No. E8). The *k*_b_ values of granules varied from 5.58 (PMRP, No. Z’1) to 24.9 (DCPA, No. E’8). These results showed that the bonding capacity of DCPA particles could not be improved after HSWG. This was related to its inherently high degree of densification and poor plastic deformation ability.

Tabletability was the ability of a powdered material to be transformed into a tablet with a certain *TS* under the applied compaction pressure [91]. For ungranulated powders, the descriptors *d* and *g* in the power model can represent the tabletability and pressure sensitivity of materials, respectively. The values of descriptor *d* ranged between 9.25 × 10^−4^ (MF, No. Z6) and 1.00 (MCC PH101, No. E15). The values of *g* varied from 0.287 (β-CD, No. E9) to 1.68 (Bistortae Rhizoma (BR), No. Z8 and MF, No. Z6). For granules, the parameters *d* were distributed between 1.07 × 10^−4^–0.517, the range of which was narrower than that of the powders. The lowest and highest value of *d* could be observed from pregelatinized starch (No. E’5) and β-CD (No. E’9), respectively. Besides, the values of *g* varied from 0.398 to 1.79. As with ungranulated powder, the granule of β-CD had the lowest *g* value of 0.398. Chuanxiong Rhizoma (No. Z7) had the highest *g* of 1.79.

Taking all materials in the material library as a whole, the compressibility of materials remained the same after HSWG. In other words, under the same level of mechanical strength, the porosity of the compact was not affected by the granulation process. The compactability of materials would decrease slightly after HSWG, which meant the *TS* of the powdered tablet was greater than that of the granular tablet at the same tablet porosity. From the *d* values and the *g* values, it could be seen that the tabletability of materials would decrease and the pressure sensitivity of materials would increase slightly after HSWG. 

#### 3.2.2. Principal Component Analysis

**Figure 4:**
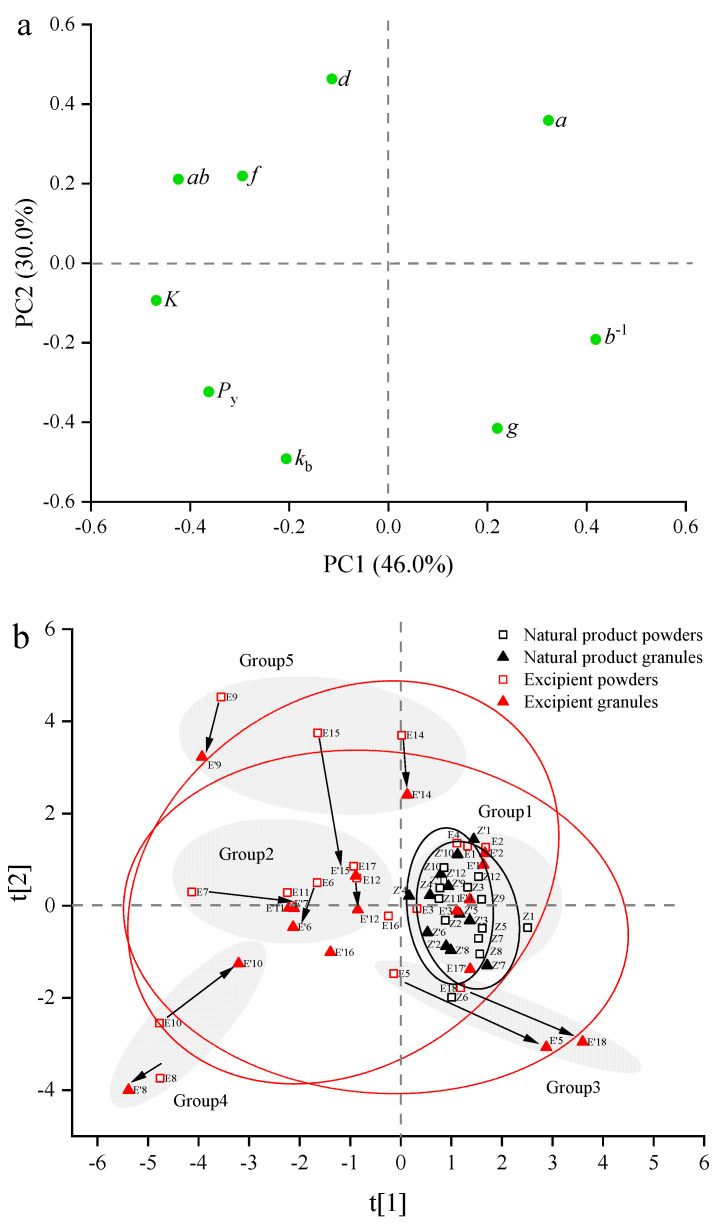
The PCA analysis of the CBCS parameters data based on the first two principal components. (**a**) the loading plot; (**b**) the score plot. (The black squares represent NPPs. The black triangles represent NPGs. The red squares represent excipient powders. The red triangles represent excipient granules. Two black circles are 95% confidence ellipses for NPPs and NPGs, respectively. Two red circles are 95% confidence ellipses for excipient powders and excipient granules, respectively. The gray shaded circles represent five groups of materials).

PCA was performed on the data matrix (size 58 × 9) consisting of 58 samples (29 ungranulated powders and 29 granules). Each sample was featured by nine CBCS parameters. The data matrix was pre-processed by the same method as described in Section 3.1.2. before PCA analysis. The first three PCs cloud explain 89.1% of the total variability, and PC1, PC2, and PC3 accounted for 46.0%, 30.0%, and 13.2%, respectively. The loading plot is shown in Figure 4a. The CBCS parameters associated with PC1 were mainly the compression resistance index *K*, the rearrangement index *ab*, the yield pressure *b*^−1^, and the fragmentation index *f*. Among them, *K*, *ab*, and *f* were located on the negative axis of PC1, while *b*^−1^ was situated on the positive axis of PC1. In particular, the parameter *ab* was close to the parameter *f*, suggesting that the particle rearrangement capacity and the particle fragmentation capacity were positively correlated within the scope of the material library. The variables contributing to PC2 were mainly the bonding index *k*_b_, the tabletability index *d* and the pressure sensitivity *g*. The descriptors *k*_b_ and *g* were distributed on the negative side of PC2, and they were on the opposite side of the descriptor *d*. This showed that materials with good bonding ability and low degree of pressure sensitivity tended to acquire good tabletability. The remaining two parameters, *a* and *P*_y_, were related to both PC1 and PC2. Parameters *a* and *P*_y_ were located at the upper right and lower left corners of the loading plot, respectively, demonstrating that materials with good plastic deformation capacity were easily compressed.

Figure 4b is the score plot of CBCS parameters. The symbols for materials and 95% confidence ellipses are the same as that in Figure 3b. It can be seen from Figure 4b that confidence areas for both excipients and NPPs are not significantly changed before and after HSWG. However, all samples can be roughly divided into five groups according to their locations in the score plot and the changing trend from powders to granules. All NPPs and four starch derivatives (i.e., CWIS, CWSS, dextrin and maltodextrin) were termed as Group 1 materials, which were mainly distributed on the positive axis of PC1 and were close to the origin. The Group 1 materials were characterized by good plastic deformation ability and weak compression resistance ability, but limited particle rearrangement and particle fragmentation. What’s more, the compression behavior of Group 1 materials did not change before and after granulation. The brittle materials, such as Lac F100 (No. E6), Lac G200 (No. E7), mannitol (No. E11), and Lac C80 (No. E12), were classified as Group 2. The Group 2 materials were situated on the negative axis of PC1, and they had extensive particle rearrangement and fragmentation, strong compression resistance ability, but poor plastic deformation ability. Generally, the Group 1 and Group 2 materials had moderate tabletability, either in the powder or granule forms. Except for Lac G200, the brittle materials mainly suffered from loss of tabletability after HSWG, as shown by the downward directions of arrows in Figure 4b. The material with the largest change in particle rearrangement ability after granulation was Lac G200, whose descriptor ab was reduced from 0.283 to 0.202. The materials of both PGS (No. E5) and CMC-Na (No. E18) belonging to Group 3 were situated at the lower right of the score plot. The changing directions of the two materials were the same, moving downward to the right. This demonstrated that PGS and CMC-Na, especially the granules of the two materials, had poor bonding ability in addition to limited particle rearrangement and particle fragmentation. A synergy of these characteristics might result in the lowest tabletability of the PGS granules (d = 1.07 × 10^−4^). Group 4 contained two materials, i.e., DCPA (No. E8) and DCP (No. E10), and they were located in the lower left of the score plot. This suggested that DCP excipients, especially DCPA, had extensive particle rearrangement and particle fragmentation, but poor plastic deformation ability, weak bonding ability and strong compressibility resistance. The weak bonding ability of DCPA might be the major reason for its poor tabletability. It was worth noting that the changing trend of the two materials from powders to granules was opposite, resulting in the different plastic deformation ability of granules. The Py of the DCP granules (386) was smaller than that of the ungranulated powders (686), while the Py of the DCPA granules (684) was larger than that of the ungranulated powders (615). β-CD (No. E9), SMCC (No. E14), and MCC PH101 (No. E15) were mainly distributed in the upper left of the score plot, and they belonged to Group 5. These materials not only had extensive particle rearrangement and particle fragmentation, but also had good bonding ability, all of which might result in good tabletability of these materials. In addition, the changing directions of the three materials from powders to granules were the same, moving from top to bottom. This indicated that the tabletability of the Group 5 materials would reduce significantly after HSWG, but the tabletability of granules were still good. There were two materials, PVPP (No. E16) and L-HPC (No. E17), that did not belong to any of the above five groups. PPVP had the largest loss of compressibility and severe reduction in plastic deformation ability, since its descriptor a was reduced from 0.870 to 0.779 and descriptor Py changed from 252 to 444, respectively. As for L-HPC, the HSWG process had the greatest impact on its particle fragmentation, because the f values were decreased from 0.221 to 0.109.

### 3.3. Change of Tabletability from Powders to Granules

#### 3.3.1. Qualitative Analysis

**Figure 5:**
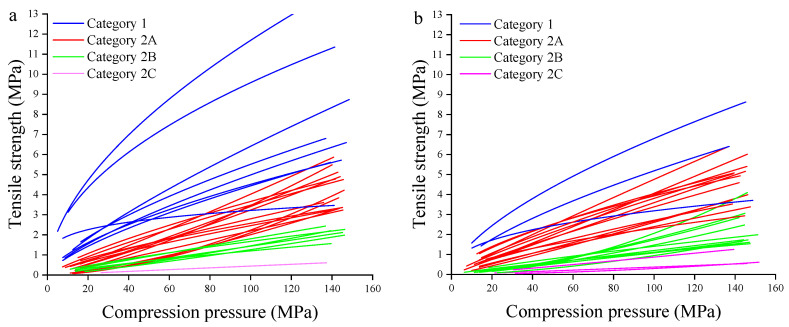
The tensile strength vs. compression pressure curves for 29 materials. (**a**) the powdered materials; (**b**) the granular materials.

The *TS* vs. compression pressure curves of 29 powdered materials are shown in Figure 5a. According to the CBCS classification criteria with respect to the tabletability, 29 powders can be divided into two categories, as shown in Table 3. Category 1 featured *d* ≥ 0.2 and 0 < *g* < 0.95. Seven pharmaceutical excipients (i.e., MCC PH101, SMCC, CWSS, CWIS, dextrin, β-CD and L-HPC) were classified into this category. These cellulose and starch derivatives excipients could produce tablets with good tabletability (*TS* ≥ 3 MPa) at the low pressures range of 20–50 MPa. Category 2 was characterized by 0.002 ≤ *d* < 0.2. Depending on the profile of the *TS*-pressure curve, it could be further subdivided into Category 2A, 2B, and 2C. In this study, 11 NPPs and four excipients belonged to the Category 2A, and these powders generally had acceptable tabletability (*TS* ≥ 2 MPa) at middle pressure range of 50–100 MPa and good tabletability at high pressure range of 100–140 MPa. One NPP (i.e., MHH, No. Z4) and five excipients were classified into the Category 2B, which meant they had acceptable *TS* at high pressure range of 100–140 MPa. Only one material, DCPA (No. E8), was classified into the Category 2C that represented unacceptable tabletability (*TS* < 2 MPa) over the investigated pressure range. 

It should be pointed out that the tabletability of materials was mainly classified according to the *d* value, while the influence of the *g* value on the curve was not considered. In this paper, it was found that the descriptor *g* had an important influence on the shape of the *TS*-pressure curve when the *d* value was lower than 0.002. For example, the *d* value of Bistortae Rhizoma (No. Z8) was 9.85 × 10^−4^, and it might be classified into the Category 3 material. However, the *g* value of the Z8 material was greater than 1.68, leading to the *TS* of obtained tablets were above 2 MPa under the pressure of 50–100 MPa and *TS* of obtained tablets were above 3 MPa under the pressure of 100–140 MPa. Therefore, the Bistortae Rhizoma powder belonged to Category 2A. This indicated that the tabletability category should be judged according to the characteristics of the *TS* vs. pressure curve when the *g* value ≥ 1.6 and the *d* value < 0.002.

**Figure 6:**
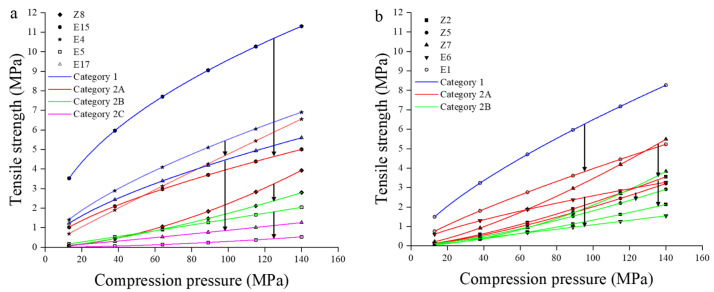
The change of tabletability for 10 materials. (**a**) the change of tabletability for 5 materials (Bistortae Rhizoma (No. Z8), maltodextrin (No. E4), pregelatinized starch (No. E5), MCC PH101 (No. E15), L-HPC (No. E17)); (**b**) the change of tabletability for 5 materials (Angelicae sinensis Radix (No. Z2), Bran-Processed Atractylodis Rhizoma (No. Z5), Chuanxiong Rhizoma (No. Z7), cold water-insoluble starch (No. E1), Lactose Flowlac 100 (No. E6)).

The *TS* vs. compression pressure curves and classification of 29 granular materials are shown in Figure 5b. The tabletability classification criteria of granules are consistent with that of powders, and the classification results are shown in Table 3. The categories of 19 materials had no change, meaning the tabletability of these materials were not significantly affected by HSWG. The other 10 materials all manifested downgrade trend. The changes of tabletability curves of these 10 materials are plotted in Figure 6. Four excipients that originally belonged to Category 1 were converted into Category 2A (i.e., CWIS, MCC PH101, maltodextrin) or 2C (i.e., L-HPC) after HSWG. The tabletability of 5 materials (i.e., Lac F100, Angelicae sinensis Radix, Bran-Processed Atractylodis Rhizoma, Chuanxiong Rhizoma, Bistortae Rhizoma) had changed from Category 2A to 2B after granulation. The category of PGS (No. E5) was transformed from Category 2B to Category 2C after granulation. Qualitative analysis demonstrated that approximately 34% of materials presented the loss of tabletability after HSWG.

#### 3.3.2. Quantitative Analysis

The reworking potential of materials varied from 13.3% (L-HPC, No. E17) to 183% (MF, No. Z6). Malkowska’s method possessed several disadvantages in assessing the change of tabletability. On the one hand, the reworking potential index had the risk of amplifying reduced tabletability of the material belonging to Category 2B and 2C. For example, the reworking potential of DCP (No. E10) was 65.8%, indicating that the material had a serious loss of tabletability. However, the tabletability category of the material before and after granulation had not changed and was maintained as Category 2B. The relative change of tabletability of DCP was −4.74% and belonged to Type Ⅱ of the following tabletability change classification system, which represented the unchanged tabletability. On the other hand, the reworking potential had a poor ability to distinguish materials. For example, the reworking potential of both Lac F100 (No. E6) and MCC PH101 (No. E15) was 40.0%, but the differences between the values of *AUC*_p_ and the *AUC*_g_ of them were 160 and 626, respectively.

**Figure 7:**
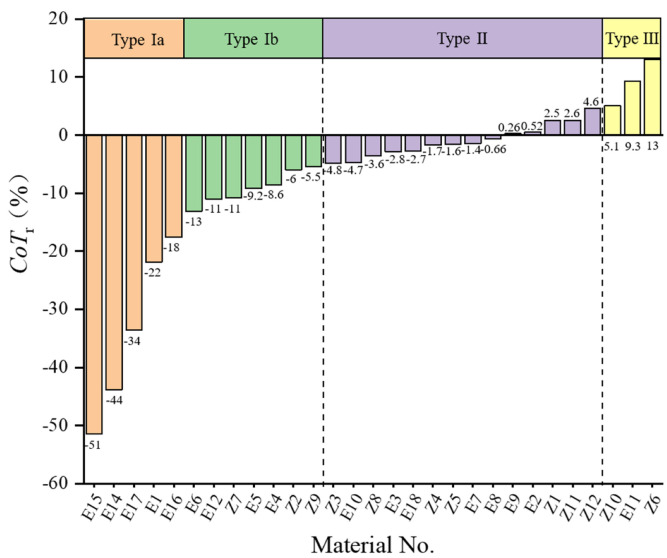
The tabletability change classification system (TCCS) and the *CoT*_r_ values for 29 materials.

In order to overcome the shortages of the reworking potential index, this study proposed a new index, as described in Section 2.6.2. According to Equation (13), the relative change of tabletability of all materials were calculated, and they varied from −51.44% (MCC PH101, No. E15) to 13.07% (MF, No. Z6) after HSWG. The relative change of tabletability of materials can be divided into three types, i.e., Type Ⅰ, Type Ⅱ, and Type Ⅲ, as shown in Figure 7. Type Ⅰ represented that a material had loss of tabletability after HSWG. According to the degree of reduced tabletability, Type Ⅰ could be further classified into two sub-types, i.e., Type Ⅰa and Type Ⅰb. The Type Ⅰa was defined as materials with large loss of tabletability, which could be represented by *CoT*_r_ < −15%. There were five excipients falling into this type (i.e., MCC PH101, SMCC, CWIS, PVPP and L-HPC). The Type Ⅰb was described as materials with moderate loss of tabletability, which was featured by −15% ≤ *CoT*_r_ < −5%. There were three NPPs (i.e., Angelicae sinensis Radix (AsR), Chuanxiong Rhizome (CxR), and Visci Herba (VH)) and four excipients (i.e., Lac F100, Lac C80, maltodextrin, and PGS) belonging to Type Ⅰb. Type Ⅱ stood for no significant change in the *CoT*_r_ of materials, which could be characterized by −5% ≤ *CoT*_r_ ≤ 5% in this study. There were seven NPPs and seven excipients belonging to the Type Ⅱ. Type Ⅲ was expressed as the increase of tabletability of materials, with the characteristic of *CoT*_r_ > 5%. One excipient (i.e., mannitol) and two NPPs (i.e., Mume Fructus and Sophorae Flavescentis Radix (SFR)) were divided into this type. 

The established tabletability change classification system (TCCS) for materials provided a new means for the initial risk evaluation of materials in the formulation design. In practice, a further evaluation could be performed in conjunction with the roles of materials used in the formulation and the tabletability categories in the CBCS. For instance, the L-HPC belonged to Type Ⅰa and its *CoT*_r_ value was −33.60%. When the L-HPC played the role of disintegrant in formulations, its proportion was often small (e.g., 2.5−5%) [92]. In such circumstances, the significant loss of tabletability of L-HPC would not be a risk since it would not alter the tabletability of the formulation to a large extent. Similarly, MCC PH101 and SMCC were commonly used fillers in tablet formulation [93,94]. The *CoT*_r_ values of MCC PH101 and SMCC were −51.44% and −43.80%, respectively, showing that the two excipients were prone to large loss of tabletability after HSWG. However, the tabletability of the two materials after wet granulation may not be a problem, since good or acceptable tablet tensile strength would be achieved. By contrast, the granules of PGS were classified as Category 2C materials after wet granulation. When the PGS was selected as diluent in the tablet formulation, a high risk would be assigned to it. 

Mannitol was a frequently used filler in the production of tablets due to its non-hygroscopic character and low drug interaction potential (inertness) [95]. The *CoT*_r_ value of mannitol was 9.32% and it belonged to Type Ⅲ. It had been reported that mannitol had increased or unchanged tabletability properties after wet granulation, which was attributed to the reduction of the primary particle size [35]. This suggested that mannitol could be used in combination with cellulose excipients having a large loss of tabletability in formulation design, so as to achieve the purpose of balancing the tabletability of the formulation [96]. Besides, mannitol was often used in immediate-release tablets or chewable tablets, due to its water-soluble and sweet taste [97]. The increased tabletability of mannitol might cause problems with disintegrating difficult of immediate-release tablets or chewing difficult of chewable tablets. This showed the increased tabletability of materials also had potential risks during tablet formulation development.

### 3.4. Comparsion of Critical Material Attributes for DC and HSWG

In order to compare the critical material attributes for DC and HSWG, a data fusion method and the PLS2 algorithm were applied. The input variables for PLS2 regression are 19 material physical properties, nine CBCS parameters, two tabletability change indexes and the compression pressure, as shown in Table 4. The tensile strength of DC tablets and the tensile strength of HSWGT tablets were taken as output variables, and were denoted by *TS*_1_ and *TS*_2_, respectively. The PLS2 algorithm possessed the advantages of using one set of latent features to predict multiple responses [98]. How the responses varied in relation to each other could be easily visualized in one loading scatter. The compression pressure was considered as the critical process parameter (CPP). In this paper, by adjusting the distance of drop of the upper punch of the tablet press, every powder or granule was compressed to a certain compression height and the corresponding compression pressure was passively recorded. Therefore, from the raw data of the *TS*–pressure relationship, it was impossible to compare the tensile strength values for both powders and granules at the same compression pressure. To address that issue, the fitted tabletability curves were used to estimate the tablet tensile strength at specified compression pressure. Within the pressure range studied, five compression pressures (i.e., 3, 5, 7, 9, 11 kN) were selected, and the tensile strength of powders (*TS*_1_) or granules (*TS*_2_) could be calculated from the fitted power equations. The distribution ranges of *TS*_1_ and *TS*_2_ was 0.16–14.13 MPa and 0.06–8.44 MPa, respectively. Each material compacted at every level of compression pressure could be treated as one observation, and each compression pressure was combined with material physical properties, CBCS parameters, and tabletability change indexes as the input variables. As a result, the total number of observations for building the PLS2 model was 145 (i.e., 29 × 5).

All data were preprocessed by mean-centering and scaling to unit variance before PLS modeling and seven-fold cross validation was used to verify the PLS model. R^2^X_cum_, R^2^Y_cum_, and Q^2^_cum_ were indexes for evaluating model performance. The Q^2^_cum_ indicated how well the model predicted new data, which was determined by cross-validation. The larger the Q^2^_cum_ value was, the better the predictive ability of the model was. Typically, 2–5 latent variables (LVs) were adequate to explain the variation well in a data set. All LVs were orthogonal to each other, and their importance descended from the first to the last LV. The diagnostics of the PLS model are displayed in Table 5. It could be seen that the first five latent variables were enough to explain the variables, and the R^2^X_cum_, R^2^Y_cum_, and Q^2^_cum_ for the PLS2 model were 73.0%, 88.8%, and 85.9%, respectively. The addition of the sixth LV could not profoundly contribute to higher R^2^ and Q^2^ values. The results revealed that the model integrating all predictors had good explanatory and predictive performance for *TS*_1_ and *TS*_2_ of tablets.

**Figure 8:**
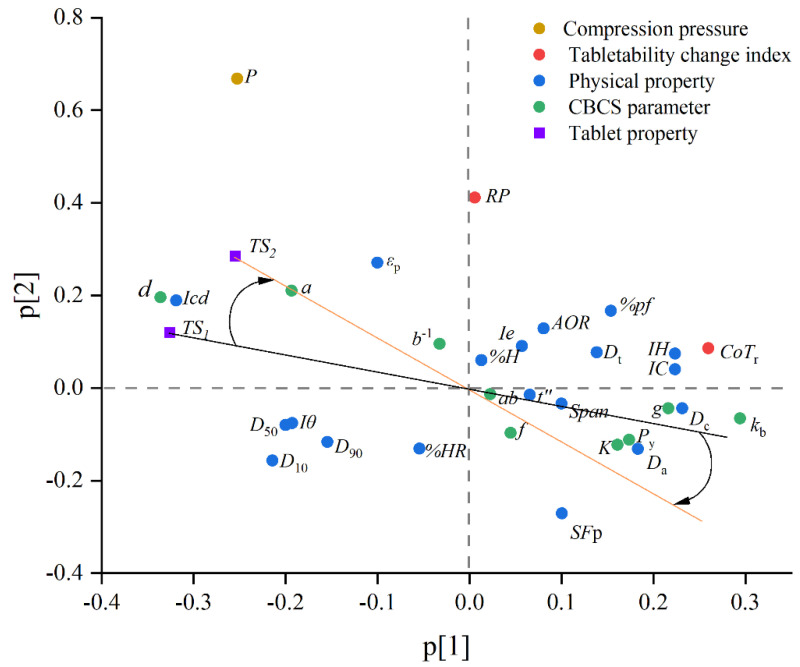
The loading plot for the PLS2 model based on the first two latent variables. (The yellow circle represents the compression pressure. The red circles represent the tabletability change indexes. The blue circles represent the physical properties. The green circles represent the CBCS parameters. The purple squares represent the tablet tensile strength. The black line represents the direction of variables associated with TS1. The yellow line represents the direction of variables associated with TS2. The arrows represent the shift in the physical properties of raw materials required by DC and HSWGT).

The first two LVs of the PLS2 model could predict 66.0% of variability in the responses data. Figure 8 gives the loading plot of Model 1 with the first latent component as the X-axis and the second latent component as the Y-axis. The *TS*_1_ and *TS*_2_ were located near to each other, implying that they are positively related. The correlation coefficient between *TS*_1_ and *TS*_2_ was 0.81, and the strong correlation between them was beneficial to driving the data projection along directions that were efficient in explaining the responses [99]. In order to identify the critical material attributes (CMAs), a line through each Y-variable and the origin was drawn. As depicted in Figure 8, the points distributed at both ends of the black line and the yellow line represent variables that are highly correlated with the *TS*_1_ and *TS*_2_ of tablets, respectively. For both *TS*_1_ and *TS*_2_, it could be found that the CBCS parameter *d* and physical property *Icd* were positively correlated with the *TS* of tablets, while the CBCS parameter *k*_b_ and physical property *D*_c_, were negatively correlated with the *TS* of tablets. This indicated that materials with strong tabletability, high cohesion index, and good bonding capacity between particles but low densification were more likely to form tablets with high *TS*, which was consistent with the results of Dai et al. [43]. The physical properties *Icd* and *D*_c_ together with the CBCS parameter *d* and *k*_b_ could be recognized as the common CMAs for DC and HSWGT. The density parameters (i.e., *D*_a_ and *D*_c_) were close to the CBCS parameters (i.e., *K*, *k*_b_ and *P*_y_), suggesting that the particle densification is not conducive to the compression and compaction of materials. The new index *CoT*_r_ and the *RP* index changed in the same direction, and the correlation coefficient of them was 0.73. The correlation coefficient between *CoT*_r_ and *TS*_1_ was −0.70, suggesting that the better the tabletability of the material, the more likely that reduced tabletability happened. The angle contained by two lines revealed the possible requirements changes of material properties. With the lines being rotated from the black to the yellow, it can be seen that the line was approaching the Kawakita *a* and *D*_a_. This implied that raw materials need to have better compressibility and deformability as well as higher porosity in order to acquire larger tensile strength during the HSWGT. In HSWG, shear force and collisions may cause consolidation of the granules and decrease in granule porosity [100]. Powders with initial high porosity and good compressibility may compensate the consolidation effect. As a result, the physical property *D*_a_ and the CBCS parameter *a* were deemed as CMAs in particular for HSWGT. 

## 4. Conclusions

In this study, the change of tabletability of pharmaceutical materials after high shear wet granulation were investigated with the help of the material library approach. Both univariate and multivariate analysis were used to compare the physical properties and the compression behaviors of ungranulated powders and granules. It was proved that HSWG had the advantages of increasing the particle size and improving the flowability of the materials. Five groups of materials with different compression behaviors could be clearly distinguished on the score plot of PCA analysis of the CBCS parameters. The HSWG process did not affect the compressibility of the materials but could decrease the compactability and tabletability of materials. The tabletability categories of 10 materials were downgraded after wet granulation. 

The reworking potential and the newly proposed index *CoT*_r_ were used to quantify the change of tabletability. The *CoT*_r_ had the advantages of mitigating the risk of amplifying the tabletability reduction for materials with weak tabletability. By summarizing the different *CoT*_r_ values of materials in the material library, the tabletability change classification system (TCCS) was successfully built. It was found that the change of tabletability of the materials could be classified into three types: loss of tabletability, unchanging tabletability, and increase of tabletability, which were defined as Type Ⅰ, Type Ⅱ, and Type Ⅲ. The materials with large and moderate loss of tabletability were further discriminated as Type Ⅰa and Ⅰb, respectively. The Type Ⅰ materials were featured by *CoT*_r_ < −5%, and 40% of materials deserved the loss of tabletability after HSWG. The Type Ⅱ stood for no significant change in the *CoT*_r_ of materials, which could be characterized by −5% ≤ *CoT*_r_ ≤ 5%, and 50% of the materials belongs to Type II. Type Ⅲ was expressed as the increase of tabletability of materials, with the characteristic of *CoT*_r_ > 5%, and 10% of the materials were divided into Type Ⅲ. The TCCS provided a means for the initial risk evaluation of materials in tablet formulation design. 

The differences in requirements of raw material properties for the two commonly used processing routes, i.e., the DC and HSWGT, were uncovered by the PLS2 modeling technique. It was found that increasing the plasticity and porosity of the starting materials was good for acquiring a high tensile strength of tablets made by the HSWGT. However, this paper only applied a material library of single materials to investigate the change of tabletability during the HSWGT, and the suggestions concluded need to be further validated by the formulation of mixtures. In order to simplify the experiment conditions, only the deionized water and ethanol were used as the wetting agents of the granulation process, and the effects of other binders as well as the process parameters on the material’s change of tabletability were not explored. These unsolved issues could be explored by means of the material library approach in future research.

## Figures and Tables

**Table 1:** The arrangements of experimental design for the high shear wet granulation process.

Run No.	Material No.	Wetting Agent	L/S Ratio (g·g^−1^)	Impeller Speed (rpm)	Addition Rate of Wetting Agent (mL·min^−1^)
1	Z1	95% ethanol	0.3	600	50
2	Z2	95% ethanol	0.3	600	50
3	Z3	95% ethanol	0.3	600	50
4	Z4	95% ethanol	0.3	600	50
5	Z5	95% ethanol	0.3	600	50
6	Z6	95% ethanol	0.3	600	50
7	Z7	95% ethanol	0.3	600	50
8	Z8	95% ethanol	0.3	600	50
9	Z9	95% ethanol	0.3	600	50
10	Z10	95% ethanol	0.3	600	50
11	Z11	95% ethanol	0.3	600	50
12	Z12	95% ethanol	0.3	600	50
13	E1	95% ethanol	0.2	300	25
14	E2	95% ethanol	0.2	300	25
15	E3	95% ethanol	0.2	300	25
16	E4	95% ethanol	0.2	300	25
17	E5	95% ethanol	0.2	300	25
18	E6	deionized water	0.1	300	25
19	E7	deionized water	0.1	300	25
20	E8	deionized water	0.1	300	25
21	E9	deionized water	0.1	300	25
22	E10	deionized water	0.2	300	25
23	E11	deionized water	0.2	300	25
24	E12	deionized water	0.3	300	25
25	E13	deionized water	0.3	300	25
26	E14	deionized water	0.5	300	25
27	E15	deionized water	0.5	300	25
28	E16	deionized water	0.6	300	25
29	E17	deionized water	0.9	300	25
30	E18	deionized water	1.3	300	25

**Table 2:** The lowest and highest values of each CBCS parameter.

Fitting Relationship	CBCS Parameter	Powders		Granules	
		The Lowest	The Highest	The Lowest	The Highest
Porosity-Pressure	*a*	0.708	1.05	0.746	1.05
	*ab*	0.0658	0.283	0.0386	0.288
	*f*	8.84 × 10^−2^	0.362	3.12 × 10^−2^	0.326
	*P*_y_	41.6	686	38.9	684
	*K*	6.35	24.5	5.87	24.9
Porosity-TS	*k*_b_	4.54	22.8	5.58	24.9
TS-Pressure	*d*	9.25 × 10^−4^	1.00	1.07 × 10^−4^	0.517
	*g*	0.287	1.68	0.398	1.79

**Table 3:** The tabletability criteria and classification results for 29 materials.

Category	Criteria	Characteristics	No. of Powders	No. of Granules
1	*d* ≥ 0.2, 0 < *g* < 0.95	Good tabletability at extreme low-pressure range (20–50 MPa)	7	3
2	0.002 ≤ *d* < 0.2 or *d* < 0.002, *g* ≥ 1.6	A: Acceptable tabletability at middle pressure (50–100 MPa) & Good tabletability at high pressure range (100–140 MPa)	15	13
	0.002 ≤ *d* < 0.05 or *d* < 0.002, *g* ≥ 1.6	B: Acceptable tabletability at high pressure (100–140 MPa)	6	10
	0.002 ≤ *d* < 0.005, *g >* 0.95 or *d* < 0.002, *g* ≥ 1.6	C: Unacceptable tabletability over the full pressure range (10–140 MPa)	1	3

**Table 4:** Description of input and output variables for the partial least squares (PLS2) model.

	Variable Type	Variables
Input	Physical property	*D*_10_, *D*_50_, *D*_90_, *Span*, *%pf*, *Iθ*, *D*_a_, *D*_c_, *D*_t_, *SF*p, *ε*_p_, *IH*, *IC*, *Ie*, *t″*, *AOR, %H*, *%HR*, *Icd*
	CBCS parameter	*A*, *b*^−1^, *ab*, *f*, *P*_y_, *K*, *k*_b_, *d*, *g*
	Tabletability change index	*RP*, *CoT*_r_
	Compression pressure	*P*
Output	Tablet property	*TS*_1_, *TS*_2_

**Table 5:** Diagnostics of the partial least squares regression model.

LVs	*R*^2^X_cum_	*R*^2^Y_cum_	*Q* ^2^_cum_
1	21.8%	52.1%	50.3%
2	41.4%	66.0%	63.0%
3	55.5%	79.5%	77.0%
4	66.0%	83.8%	80.6%
5	73.0%	88.8%	85.9%
6	78.4%	90.0%	85.5%

## Data Availability

The data presented in this study are available in Supplementary Materials.

## Supplementary Materials

The following supporting information can be downloaded at: https://www.mdpi.com/article/10.3390/pharmaceutics14122631/s1. Supplementary Materials S1: Table S1. The information of NPPs and pharmaceutical excipients including names, abbreviations, batch numbers and suppliers. Supplementary Materials S2: Spreadsheet S1. The physical properties, model fitting results, CBCS parameters and tabletability classification for all materials.

## Abbreviations

**Abbreviations**	**Full Name**
*A*_max_	Maximum change of tabletability
*AOR*	Angle of repose
APIs	Active pharmaceutical ingredients
*AUC*_g_	The area under the tensile strength vs. compression pressure profile of granules
*AUC*_max_	The maximum area under the curve
*AUC*_min_	The minimum area under the curve
CBCS	Compression behavior classification system
CMAs	Critical material attributes
*C*_max_	Degree of compression at maximal pressure
*CoT*_r_	Relative change of tabletability
CPP	Critical process parameter
*D*_a_	Bulk density
*D*_c_	Tapped density
DC	Direct compression
DG	Dry granulation
*D*_t_	True density
HSWG	High shear wet granulation
HSWGT	High shear wet granulation tableting
*%H*	Hygroscopicity
*%HR*	Loss on drying
*IC*	Carr index
*Icd*	Cohesion index
*Ie*	Inter-particle porosity
*IH*	Hausner ratio
*Iθ*	Homogeneity index
LVs	Latent variables
MCS	Manufacturing classification system
NPGs	Natural product granules
NPPs	Natural product powders
OSD	Oral solid dosage
PC	Principal component
PCA	Principal component analysis
*%pf*	Percentage of particles measuring < 50 μm
PLS	Partial least squares
R^2^	Determinant coefficient
RH	Relative humidity
RMSE	Root mean square error
RP	Reworking potential
SF	Solid fraction
SKH	Shapiro-Konopicky-Heckel
*t’’*	Flowability
TCCS	Tabletability change classification system
TS	Tensile strength
WG	Wet granulation
