# Supplementary material for: Using a Material Library to Understand the Change of Tabletability by High Shear Wet Granulation
Source: Pharmaceutics. 2022 Nov 28;14(12):2631. doi: 10.3390/pharmaceutics14122631 (PMC9783360; doi:10.3390/pharmaceutics14122631)
Supplement: Supplementary file 1 [file pharmaceutics-14-02631-s001.zip › Supplementary materials S1.pdf]

Table S1. The information of NPPs and pharmaceutical excipients including names, abbreviations, batch numbers and suppliers

Materials type	No.	Name	Abbreviation	Batch No.	Supplier
NPPs	Z1	*Polygoni Multiflori Radix Praeparata*	PMRP	PYP170920-526124-22A	Beijing Tcmages Pharmaceutical Co., Ltd.
	Z2	*Angelicae sinensis Radix*	AsR	YP170122-748304-02A	Beijing Tcmages Pharmaceutical Co., Ltd.
	Z3	*Asari Radix Et Rhizoma*	ARER	180427-113200-38A	Beijing Tcmages Pharmaceutical Co., Ltd.
	Z4	*Menthae Haplocalycis Herba*	MHH	J180627-611300-13A	Beijing Tcmages Pharmaceutical Co., Ltd.
	Z5	*Bran-Processed Atractylodis Rhizoma*	BPAR	PYP180209-067400-07A	Beijing Tcmages Pharmaceutical Co., Ltd.
	Z6	*Mume Fructus*	MF	J180606-672600-07A	Beijing Tcmages Pharmaceutical Co., Ltd.
	Z7	*Chuanxiong Rhizoma*	CxR	YP170122-748411-01A	Beijing Tcmages Pharmaceutical Co., Ltd.
	Z8	*Bistortae Rhizoma*	BR	P190116-264400-03A	Beijing Tcmages Pharmaceutical Co., Ltd.
	Z9	*Visci Herba*	VH	P190226-134100-01A	Beijing Tcmages Pharmaceutical Co., Ltd.
	Z10	*Sophorae Flavescentis Radix*	SFR	180209-046300-07A	Beijing Tcmages Pharmaceutical Co., Ltd.
	Z11	*Cinnamomi Cortex*	CC	YP180209-526100-07A	Beijing Tcmages Pharmaceutical Co., Ltd.
	Z12	*Buddlejae Flos*	BF	P190424-638300-02A	Beijing Tcmages Pharmaceutical Co., Ltd.
Excipients	E1	Cold water-insoluble starch	CWIS	Y16S10F97679	Shanghai yuanye Bio-Technology Co., Ltd.
	E2	Cold water-soluble starch	CWSS	Y26S10F98934	Shanghai yuanye Bio-Technology Co., Ltd.
	E3	Dextrin	/	T14A10295117	Shanghai yuanye Bio-Technology Co., Ltd.
	E4	Maltodextrin DE20	MD	2019101751	Liaoning Luquan Pharmaceutical Technology Co., Ltd.
	E5	Pregelatinized starch	PGS	20200628	Huzhou Prospect Pharmaceutical Co., Ltd.
	E6	Lactose Flowlac® 100	Lac F100	L101501320	Molkerei Meggle Wasserburg GmBH & Co. KG
	E7	Lactose Granulac® 200	Lac G200	L1535	Molkerei Meggle Wasserburg GmBH & Co. KG
	E8	Dibasic calcium phosphate anhydrous	DCPA	D03424A	TechTex-Verlag GmbH & Co. KG
	E9	β-cyclodextrin	β-CD	200311	Liaoning Luquan Pharmaceutical Technology Co., Ltd.
	E10	Dibasic calcium phosphate	DCP	C93226A	TechTex-Verlag GmbH & Co. KG
	E11	Mannitol	/	122000024	SPI Pharmaceutical Technology Co., Ltd.
	E12	Lactose Cellactose® 80	Lac C80	L100461420	Molkerei Meggle Wasserburg GmBH & Co. KG
	E13	Calcium phosphate	CaP	C99038A	TechTex-Verlag GmbH & Co. KG
	E14	Silicified microcrystalline cellulose	SMCC	ZC20501	SPI Pharmaceutical Technology Co., Ltd.
	E15	Microcrystalline cellulose PH101	MCC PH101	L1545	Shanghai Changwei Pharmaceutical Excipients Technology Co., Ltd.

E16	Polyvinylpolypyrrolidone XL-10	PPVP XL-10	0002493602	Ashland_Aqualon, Pharmaceutical Co., Ltd.
E17	Low-substituted hydroxypropyl cellulose LH11	L-HPC LH11	20210113	Huzhou Prospect Pharmaceutical Co., Ltd.
E18	Croscarmellose sodium	CMC-Na	10082Fo	DFE Pharma GmbH & Co. KG
